# Mn_3_O_4_ nanoparticle-decorated hollow mesoporous carbon spheres as an efficient catalyst for oxygen reduction reaction in Zn–air batteries†

**DOI:** 10.1039/d0na00428f

**Published:** 2020-06-19

**Authors:** Yingjie He, Drew Aasen, Haoyang Yu, Matthew Labbe, Douglas G. Ivey, Jonathan G. C. Veinot

**Affiliations:** Department of Chemistry, University of Alberta 11227 Saskatchewan Drive Edmonton Alberta T6G 2G2 Canada jveinot@ualberta.ca; Department of Chemical and Materials Engineering, University of Alberta 9211 116 St Edmonton Alberta T6G 1H9 Canada

## Abstract

Hybrids of Mn_3_O_4_ nanoparticles and hollow carbon spheres prepared from templated pyrolysis of polydopamine were assembled *via* a straightforward sonication procedure. The resulting hybrids exhibit excellent catalytic activity toward the oxygen reduction reaction (ORR) in prototype Zn–air batteries. Impressively, these catalysts exhibit higher discharge potential and exceptional stability when compared to commercial Pt–Ru catalysts while simultaneously showing comparable onset potential and maximum current density.

## Abstract 2

Hybrids of hollow mesoporous carbon and Mn_3_O_4_ nanoparticles exhibit excellent catalytic activity on oxygen reduction reaction (ORR) in Zn–air battery.

## Graphical abstract

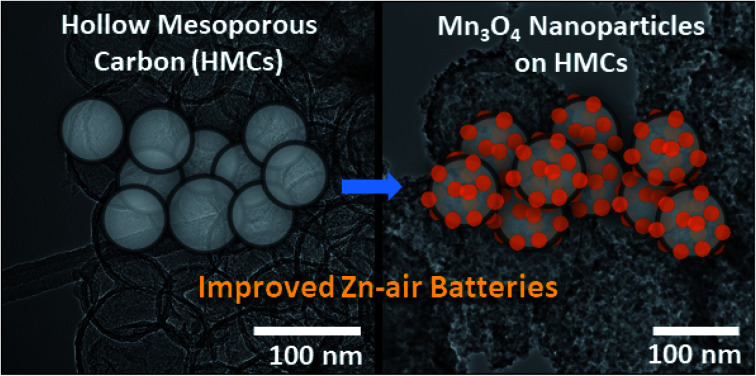

## Introduction

Society's seemingly insatiable demand for energy and the associated environmental impact of fossil fuel use have led to calls for a global transition to renewable energy sources that include wind and solar power.^1^ Despite maturing technologies and declining cost, the intrinsic intermittency of renewable energy production remains a challenge for large scale commercialization and implementation.^1,2^ One approach toward mitigating this important issue is employing energy storage units that can provide better alignment of energy supply and demand timing.^3,4^ Clearly, developing efficient and cost-effective cyclable energy storage technologies, like batteries, is of nascent importance.^5^

Among the many technologies that have appeared, metal–air batteries have attracted substantial attention in part because of their comparatively high energy density.^6–10^ Aqueous Zn–air batteries are particularly attractive for stationary energy storage because they are based upon earth abundant, non-toxic elements (*i.e.*, Zn) and have a high theoretical energy density (1086 kW kg^−1^) which is four times that of current Li-ion batteries.^11^ Despite these advantages, further advances are required if Zn–air batteries are to realize their full potential. The most substantial benefits will come from the development of efficient precious metal-free catalysts that facilitate reactions at the air electrode.^12^

**Fig. 1:**
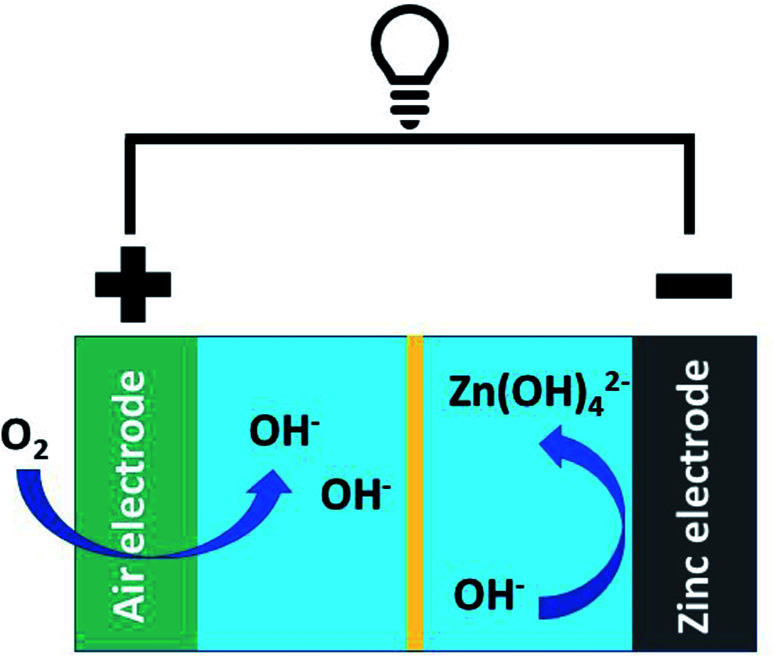
A general schematic of a Zn–air battery and its associated processes.

The oxygen reduction and evolution reactions (*i.e.*, ORR and OER, respectively) occur at the air electrode and are crucial to battery performance (Fig. 1). When the battery is discharging, the ORR reaction is active and oxygen is reduced to form zincate anions at the Zn electrode. Upon charging, this process is reversed and oxygen is regenerated *via* the OER. While ORR is thermodynamically favourable under operational conditions, both the ORR and OER are kinetically hindered.^13^ As a result, electrocatalysts are essential to realizing functional/cyclable batteries. Conventional ORR and OER catalysts rely on precious metals and their oxides.^14^ The high cost of these metals/metal oxides potentially limits the economic viability of their large-scale application.

Precious-metal free systems are being aggressively explored in efforts to find alternative, efficient catalysts. Of late, a variety of carbon nanomaterials (*e.g.*, N-doped carbon nanotubes (CNTs), carbon nanoribbons, and graphene nanosheets) have shown excellent ORR and/or OER catalytic activity.^15–18^ Transition metal oxide nanoparticles (*e.g.*, MnO_*x*_, CoO_*x*_) also exhibit promising performance.^19,20^ There have even been reports of synergistic effects when these materials are combined, however its chemical origin was not identified.^21^ Previously we demonstrated that N-doped hollow mesoporous carbon (HMC) nanospheres for their catalytic activity.^22^

Here, we report a new precious-metal free hybrid that combines the favourable ORR properties of N-doped HMCs with those of earth abundant Mn_3_O_4_ nanoparticles. The hybrid exhibits enhanced catalytic ORR performance when compared with the individual components, as well as benchmark Pt–RuO_2_ catalysts. Further adding to the appeal of this new catalyst, its stability exceeds that of Pt–RuO_2_ standards when incorporated into prototype Zn–air batteries.

## Experimental section

### Chemicals

Dopamine hydrochloride, pluronic F127, tris(hydroxymethyl)amino-methane (Tris), tetraethoxysilane (TEOS) (≥99%), 2-propanol, and anhydrous ethanol (≤0.005% water) were obtained from Sigma-Aldrich. Ammonium hydroxide (NH_4_OH; 28–30%) was acquired from Caledon Laboratory Chemicals. Hydrofluoric acid (HF, electronic grade, 48–50%) and sodium hydroxide (NaOH, pellets) were purchased from Fisher Scientific. Manganese acetate tetrahydrate (Mn(CH_3_COO)_2_·4H_2_O, powder) was obtained from Matheson Colman & Bell. Pt–Ru powder (30% Pt and 15% Ru on carbon black) and Nafion solution (D521, 5% w/w in water) were purchased from Alfa Aesar. Teflon-coated porous carbon paper (SGL 39BC gas diffusion layer; GDL) was acquired from the Fuel Cell Store. All reagents were used as received unless otherwise specified.

### Synthesis of Stöber SiO_2_ nanoparticles (SiO_2_ NPs)

Stöber SiO_2_ NPs were prepared using a modified literature procedure.^23^ Briefly, TEOS (3.57 mL, equiv. 16.1 mmol) was added to a mixture of deionized water (80 mL) and anhydrous ethanol (156 mL), followed by the addition of NH_4_OH (8.0 mL). The reaction mixture was stirred at 500 rpm for 3 h at room temperature (25 °C). Subsequently, the SiO_2_ NPs were isolated upon centrifugation at 12 000 rpm for 30 min. They were then re-dispersed in anhydrous ethanol and purified by centrifugation. The dispersion/isolation process was repeated twice. The purified SiO_2_ NPs were dried in ambient conditions (in air at 25 °C) for 16 h, after which they were transferred to a sealed glass vial and stored at room temperature (25 °C) and under ambient light until needed.

### Synthesis of SiO_2_@C nanoparticles (NPs)

The SiO_2_ NPs (0.700 g) were dispersed in 187 mL deionized water with exposure to a bath sonicator for 10 min. Triblock copolymer pluronic F127 (0.350 g), Tris (0.210 g), and dopamine hydrochloride (0.700 g) were added as powders to the SiO_2_ NP suspension. The mixture was magnetically stirred at 500 rpm for 24 h at room temperature (25 °C). The resulting polydopamine coated SiO_2_ NPs (SiO_2_@PDA) were isolated *via* centrifugation at 12 000 rpm for 30 min. Subsequently, the SiO_2_@PDA were re-dispersed in DI water with sonication and recovered *via* centrifugation. This process was repeated twice, replacing water with anhydrous ethanol. The purified SiO_2_@PDA was dried in air at 25 °C for 16 h. The SiO_2_@PDA was then transferred to a quartz boat, placed in a standard tube furnace and carbonized under flowing Ar atmosphere. The furnace was heated to 400 °C (heating rate of 1 °C min^−1^) and maintained at 400 °C for 2 h before the heating to 800 °C (heating rate of 5 °C min^−1^) for 3 h. Finally, the furnace was cooled to 25 °C and SiO_2_@C NPs were obtained as a black solid.

### Synthesis of hollow mesoporous carbon spheres (HMCs)

The as-synthesized SiO_2_@C (0.600 g) was dispersed in anhydrous ethanol (6.0 mL) and DI water (6.0 mL) *via* magnetic stirring (10 min at 500 rpm) followed by sonication (10 min) in a polyethylene terephthalate (PET) beaker. HF (49%, 6.0 mL) was added to the mixture, which was magnetically stirred at 25 °C for 1 h. The HF dissolved the SiO_2_ core without damaging the carbon shell, yielding hollow mesoporous carbon spheres (HMCs). The HMCs were collected *via* vacuum filtration using a Teflon funnel and filter flask and washed with 60 mL of DI water and anhydrous ethanol (**Caution**! HF must be handled with extreme care and in accordance with local regulations).

### Synthesis of Mn_3_O_4_@HMC

The hybrid material was synthesized according to a modified literature procedure.^21^ The purified HMCs (50 mg) were dispersed in anhydrous ethanol (10.0 mL) and magnetically stirred for 10 min at 500 rpm. Subsequently, Mn(CH_3_CO_2_)_2_·4H_2_O (250 mg) and NaOH (100 mg) were added to the HMC ethanol suspension. The mixture was transferred to a sealed glass reaction vessel and exposed to a bath sonicator for 2.5 h. The water bath was replenished with cold water and the sonication was continued for an additional 2.5 h. The final product (Mn_3_O_4_@HMC) was isolated *via* centrifugation at 12 000 rpm for 30 min. It was then re-suspended in anhydrous ethanol and centrifuged. The process was repeated three times. The centrifuged Mn_3_O_4_@HMC was again re-dispersed in anhydrous ethanol and filtered *via* vacuum filtration.

### Synthesis of freestanding Mn_3_O_4_

Freestanding Mn_3_O_4_ NPs were synthesized for comparison. The preparation was identical to that for Mn_3_O_4_@HMC; however, no HMC was added to the reaction mixture. Mn(CH_3_CO_2_)_2_·4H_2_O (250 mg) and NaOH (100 mg) were added to ethanol (10.0 mL), followed by sonication (5 h) in a bath sonicator.

### Material characterization

Fourier-transform infrared (FTIR) spectra were obtained with a Nicolet 8700 (Thermo Fischer, USA) FT-IR spectrometer. FTIR samples were prepared by mixing the material of interest (∼1 mg) and potassium bromide fine powder (KBr, 150 mg). The powder mixture was then pressed into a pellet using a hand press (Specac Ltd).

Scanning electron microscopy (SEM) was performed using a Zeiss Sigma 300 VP-FESEM (accelerating voltage of 5–20 kV) equipped with secondary and backscattered electron detectors and an in-lens detector. Samples were prepared by placing a drop of ethanol suspension of the material of interest onto an Al stub and was subsequently air dried for 1 h at room temperature (25 °C). Transmission electron microscopy (TEM) was performed with a JEOL JEM-ARM200CF TEM/STEM (accelerating voltage of 200 kV) equipped with energy dispersive X-ray (EDX) spectrometer. High resolution TEM (HRTEM) images were processed using Gatan Digital Micrograph software (Version 3.22.1461.0) and ImageJ (Version 1.52R). Selected area electron diffraction (SAED) was performed on a JEOL 2010 TEM. TEM samples were prepared by dispersing the purified sample in anhydrous ethanol, which was then drop cast onto a holy/lacey carbon coated Cu grid (Electron Microscopy Inc.) and dried under vacuum for at least 16 h. At least 300 nanoparticles were used for determination of thickness and diameter of the purified materials. EDX maps of several representative regions were also obtained.

X-ray photoelectron spectroscopy (XPS) was performed with a Kratos Axis 165 Ultra X-ray spectrometer operating in energy spectrum mode under ultra-high vacuum. A monochromatic Al K source (*λ* = 8.34 Å) was used as the X-ray source. The samples were prepared by drop-casting an ethanol suspension of material of interest onto a Cu foil that was air dried for at least 16 h at room temperature (25 °C). The take-off angle was 90°. CasaXPS software (VAMAS) was used to analyse the obtained data. In general, a Shirley-type background was subtracted.^24^ The spectra were calibrated by setting the deconvoluted adventitious C 1s peak to 284.8 eV.

Powder X-ray diffraction (PXRD) was conducted by placing purified samples on a zero-background Si wafer. PXRD patterns were collected using a Rigaku Ultima IV XRD system equipped with a Cu Kα radiation source.

Nitrogen adsorption–desorption isotherms were obtained with a Quantachrome Autosorb-iQ-XR system at −196 °C. Before measurements, samples were outgassed at 150 °C under vacuum for 3 h. Data was analysed using Brunauer–Emmett–Teller (BET) theory. The BET specific surface area was extrapolated from the linear region of the BET graph and the total pore volume was obtained from the data point at around *P*/*P*_0_ = 0.992.

### Electrochemical testing

The electrochemical performance of candidate catalysts was evaluated using linear sweep voltammetry (LSV). Measurements were performed in O_2_ saturated 1 M KOH with a potentiostat (VSP-100) using a three-electrode cell and at a scan rate of 5 mV s^−1^. The KOH solution was purged with O_2_ gas and was stirred constantly during testing. The working electrode was prepared as follows. An ethanol suspension was prepared by dispersing 50 mg of sample of interest in 15 mL of anhydrous ethanol and 1 mL of 5% Nafion. A pre-cut piece of GDL (circular, diameter = 4.5 cm) was soaked and sonicated in the ethanol suspension for 20 min, before it was dried in air at 25 °C for 15 min. Subsequently, the ethanol suspension (3 mL) was passed through the GDL *via* vacuum filtration, producing an impregnated GDL with a mass loading of ∼2 mg cm^−2^. For comparison, a Pt–Ru sample was prepared by spray coating an ink consisting of Pt–Ru/C (50 mg), deionized water (1.00 mL), ethanol (2.00 mL), and 5% Nafion (0.100 mL) onto the GDL. The impregnated or sprayed GDL samples were used as the working electrode. A platinum wire and Hg/HgO (0.098 V *vs.* SHE) were used as counter electrode and reference electrode, respectively. The reported current densities were normalized to the exposed surface area of the working electrode. All reported potentials were *IR* compensated (*R*_u_ = 2–4 Ω).

### Prototype Zn–air battery assembly

Prototype Zn–air battery testing was performed in both vertical and horizontal home-made cells. The vertical cell had a two-electrode setup whereas the horizontal cell had a tri-electrode configuration (decoupled electrodes for ORR and OER).^25^ In both configurations, the electrolyte consisted of 6 M KOH and 0.25 M ZnO. In the vertical cell, zinc sheet (8 g) and catalyst loaded GDL were used as zinc and air electrodes. Discharge rate test and power density curves were obtained using this configuration. The horizontal cell consisted of zinc sheet (8 g), catalyst loaded GDL, and Ni foam as zinc, ORR, and OER electrodes respectively. Electrochemical impedance spectra (EIS) and discharge and charge cycling were performed using this setup. For comparison, Pt–Ru spray-coated GDL was used as the air electrode in the vertical cell and ORR electrode in the horizontal cell. Discharge and charge cycling were performed at 20 mA cm^−2^ and 30 min per cycle.

### Rotating disk electrode (RDE) testing

Cyclic voltammetry (CV) and linear sweep voltammetry (LSV) were also performed using a glassy carbon rotating disk electrode (RDE) (Pine instruments Co). An ink was prepared by combining 4 mg of the Mn_3_O_4_@HMC, 0.250 mL Nafion solution, and 0.750 mL 2-propanol. The ink was sonicated for 1 h in a bath ultrasonicator filled with ice-water. The dispersed ink was immediately drop-cast (0.005 mL aliquot) onto a polished and cleaned RDE electrode. The electrode was then dried under a heat lamp (20 W) for 1 h (mass loading 0.1 mg cm^−2^). A three-electrode cell was used, with the catalyst coated RDE, Pt coil, and Hg/HgO as working, counter, and reference electrodes, respectively. 0.1 M KOH aqueous solution was used as the electrolyte. CV and LSV were performed at a scan rate of 20 and 5 mV s^−1^, respectively, in O_2_-saturated electrolyte. O_2_-saturated electrolytes were prepared by purging 0.1 M KOH with high purity O_2_ for at least 25 min at a rate of 0.04 standard litres per minute (SLPM).

## Results and discussion

HMCs were prepared using a method developed in our laboratory.^22^ Silica (SiO_2_) nanoparticles (NPs) were synthesized *via* the Stöber method and used as a sacrificial template. The as-synthesized SiO_2_ NPs were coated with dopamine and pluronic F127. The coating was polymerized under basic conditions, after which the polymer shell was carbonized. The SiO_2_ core was removed *via* alcoholic hydrofluoric acid (HF) etching to yield the HMCs on/in which, Mn_3_O_4_ NPs were deposited.^21^

**Fig. 2:**
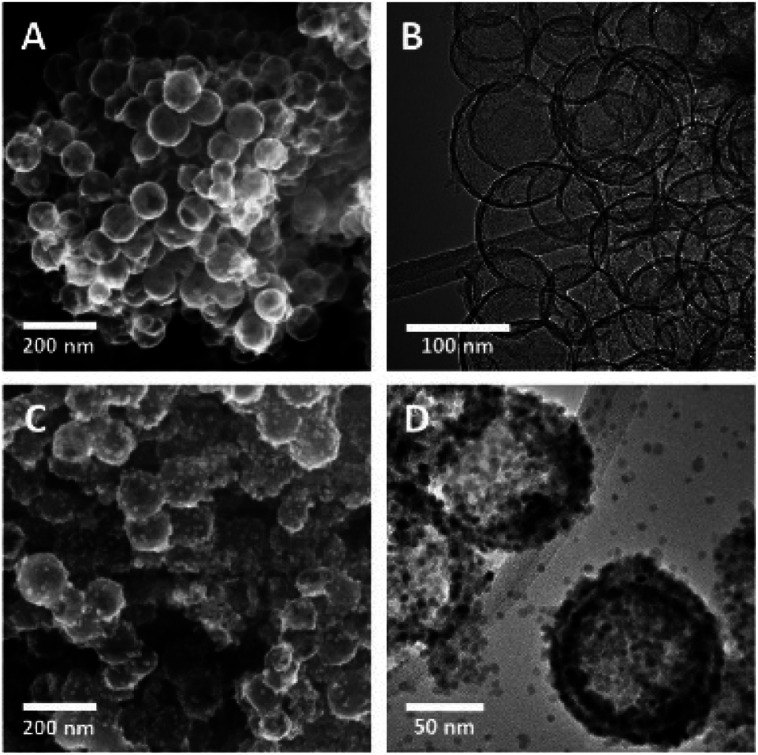
Representative SEM secondary electron (SE) (A) and TEM bright field (BF) (B) images of HMC; SEM SE (C) and TEM BF (D) images of Mn_3_O_4_@HMC.

**Fig. 3:**
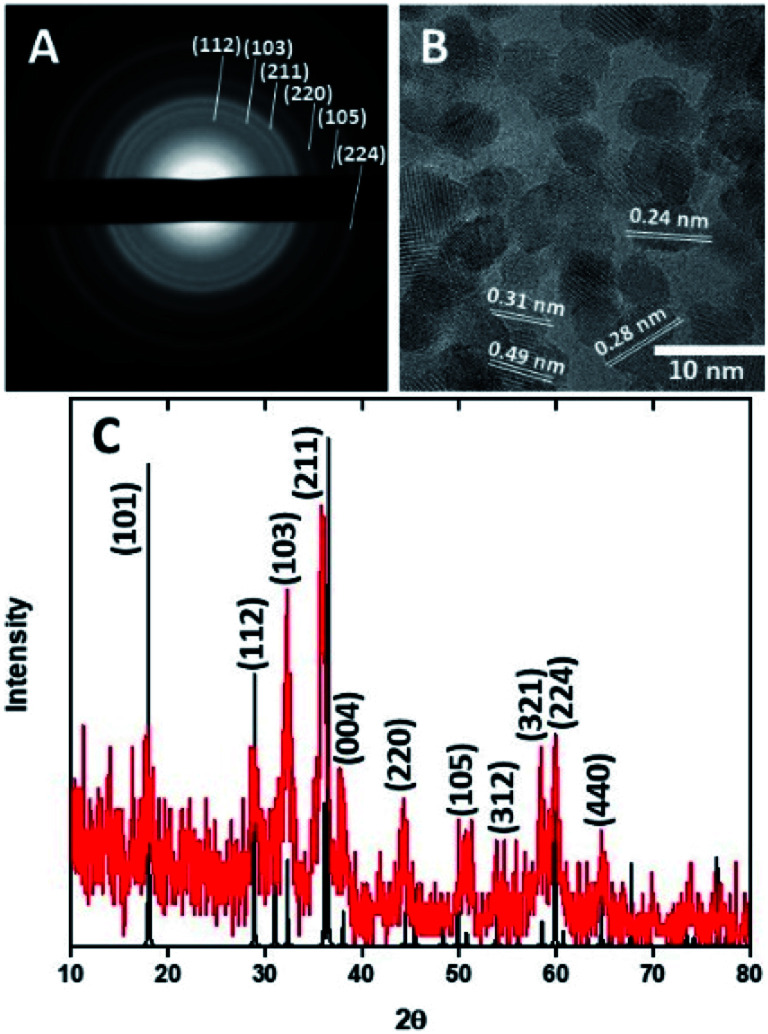
Selected area electron diffraction pattern (A), HRTEM image (B), and PXRD pattern (C) for Mn_3_O_4_@HMC.

Fig. 2A and B show electron microscopy images of as-synthesized HMCs. It is clear that HF etching removes the SiO_2_ core to yield a hollow structure that retains a spherical morphology with a diameter of 98 ± 20 nm and a shell thickness of 3.8 ± 0.4 nm (Fig. 2B). After addition of Mn(CH_3_CO_2_)_2_ and NaOH followed by 5 h of sonication, the morphology of the HMCs remained intact (Fig. 2C). The SEM image reveals a rough surface on the modified HMCs that presumably arises from the deposition of NPs. Higher magnification inspection using TEM (Fig. 2D) reveals Mn_3_O_4_ NPs on the surfaces and within the HMCs. The average NP size was determined to be 3.8 ± 0.5 nm (Fig. S6†). The crystallinity of the Mn_3_O_4_ NPs is clear from the selected area electron diffraction (SAED) pattern (Fig. 3A), which shows rings with *d*-spacings of 0.312, 0.282, 0.251, 0.204, 0.182, and 0.155 nm, that we confidently assigned to (112), (103), (211), (220), (105), and (224) planes, respectively, of hausmannite Mn_3_O_4_ (JCPDS card 24-0734).^25,26^ These *d*-spacings were also directly observed *via* HRTEM (Fig. 3B). PXRD analysis (Fig. 3C) further confirms the reflections are readily indexed to hausmannite Mn_3_O_4_.^26^

**Fig. 4:**
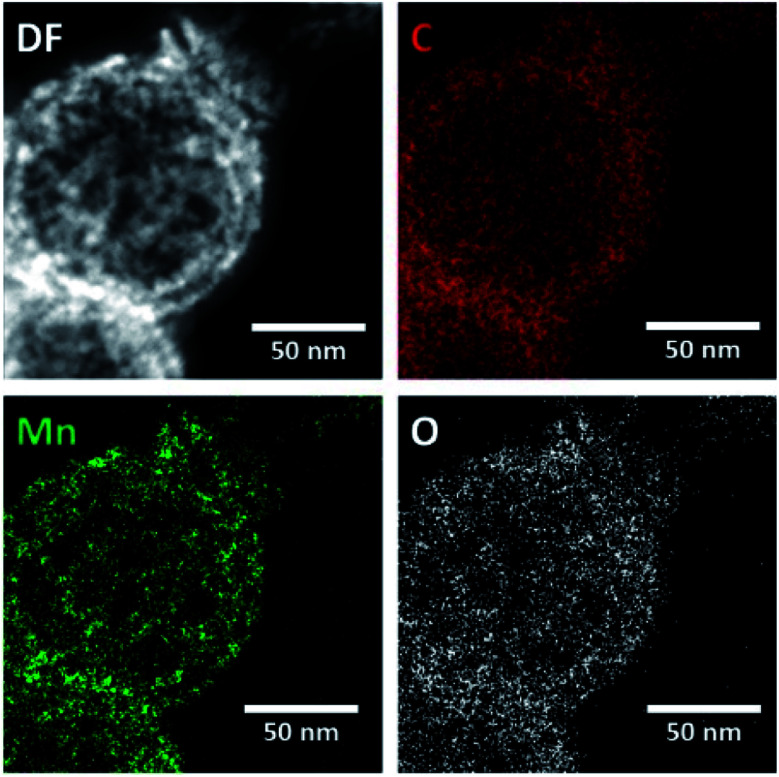
EDX mapping of Mn_3_O_4_@HMC showing uniform distribution of Mn and O.

EDX mapping of the HMCs (Fig. S1†) shows the N signal overlaps with the bright field image, which is consistent with N being incorporated into the carbon matrix. The oxygen present in the carbon matrix can be reasonably attributed to residual oxygen remaining after the pyrolysis of polydopamine. Fig. 4 shows EDX mapping for the Mn_3_O_4_@HMC. The C, Mn, and O signals all overlap with the STEM HAADF image. EDX analysis shows that the NPs are uniformly distributed and have a composition consistent with Mn_3_O_4_.

**Fig. 5:**
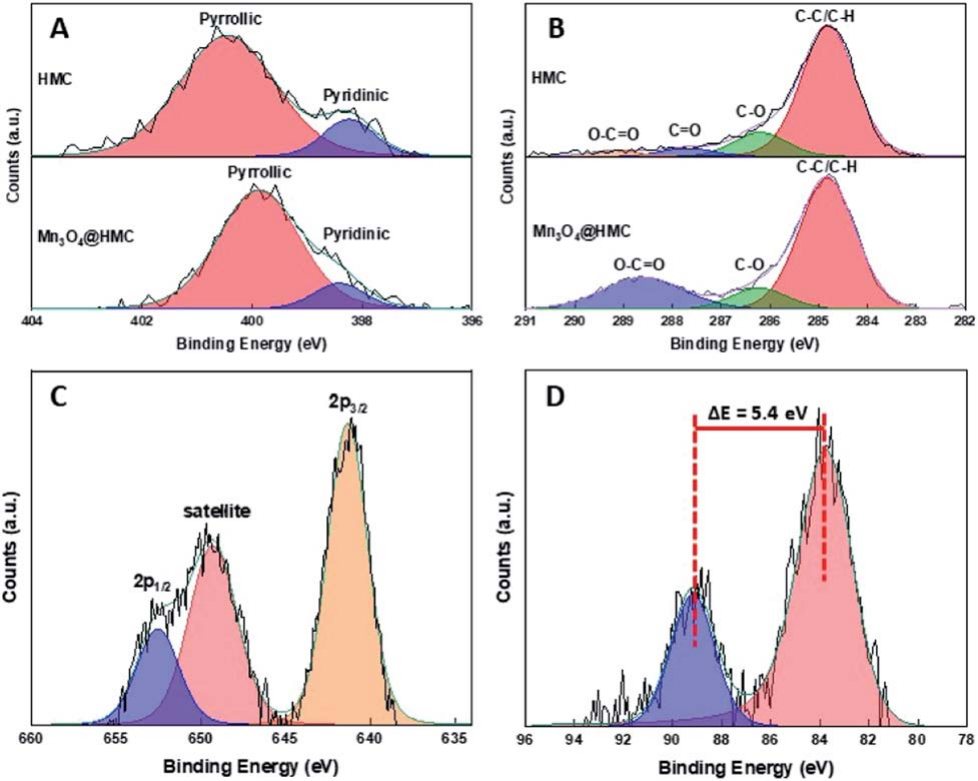
High resolution XP spectra for (A) N 1s, (B) C 1s, (C) Mn 2p, and (D) Mn 3s regions.

The Mn_3_O_4_@HMC hybrid was also characterized using XP and FTIR spectroscopy. As expected, the XP survey spectrum (Fig. S2†) shows emissions associated with Mn, C, N, and O. The deconvoluted N 1s XP spectrum (Fig. 5A) for HMC shows pyridinic and pyrrolic N at 398.2 eV and 400.5 eV, respectively. After incorporation of Mn_3_O_4_, the pyridinic N emission did not shift, while the pyrrolic N peak shifted from 400.5 eV to 399.9 eV. This observation can reasonably be attributed to pyrrolic N interacting with the Mn_3_O_4_ NPs and is the subject of ongoing investigation in our laboratory. In the deconvoluted C 1s spectrum (Fig. 5B), the C=O emission shifted to a higher binding energy and merged with the O–C=O feature, consistent with the C=O species also interacting with the Mn_3_O_4_ surface through the oxygen. The deconvoluted Mn 2p spectrum (Fig. 5C) of the hybrids shows Mn 2p_3/2_ at 641.4 eV, confidently assigned to Mn_3_O_4_.^27^ A satellite peak corresponding to Mn_3_O_4_ was also observed at 649.2 eV. The Mn 3s spectrum (Fig. 5D) shows a signature 5.4 eV splitting of the 3s emission that is commonly attributed to the presence of Mn^3+^.^27–29^

**Fig. 6:**
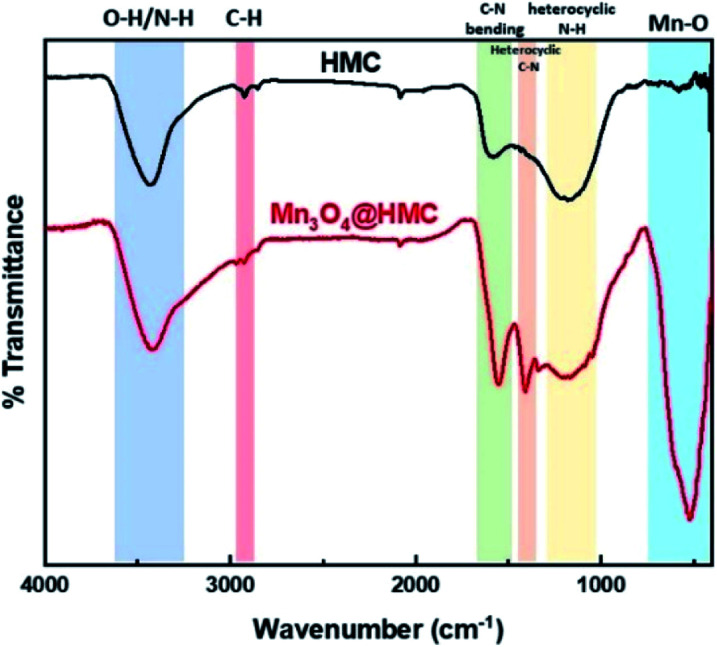
FTIR spectra for HMC and Mn_3_O_4_@HMC.

FTIR spectra for HMC and Mn_3_O_4_@HMC are shown in Fig. 6. The HMC spectrum shows an absorption at 3434 cm^−1^ that is attributed to surface O–H and N–H stretching.^30^ The feature at 2925 cm^−1^ arises from C–H stretching.^30^ Features at 1585 and 1435 cm^−1^ correspond to C–N bending and heterocyclic stretching, respectively, and the feature at 1172 cm^−1^ is assigned to heterocyclic N–H in-plane deformation breathing.^30^ In addition to all the HMC spectral features, the Mn_3_O_4_@HMC spectrum shows an absorption at 524 cm^−1^ corresponding to Mn–O bonding.^31^ The C–N bending and heterocyclic stretching features in the HMC spectrum are qualitatively sharper and located at lower energy (*i.e.*, 1556 and 1410 cm^−1^, respectively) for the hybrid, which is consistent with HMC-NP interactions suggested by the N-region of the XP spectra (Table 1).

**Table 1:** HMC and Mn_3_O_4_@HMC textural properties

Catalyst	BET surface area (m^2^ g^−1^)	Pore diameter (nm)	Pore volume (cm^3^ g^−1^)
Pristine HMC	498	29	3.33
Mn_3_O_4_@HMC	201	24	1.28

The textural properties of the pristine HMC and Mn_3_O_4_@HMC were evaluated by nitrogen sorption analysis. BET surface area, pore diameter, and pore volume were acquired. Isotherms (Fig. S3 and S4†) show a distinct hysteresis loop was observed at high relative pressure for the pristine HMCs giving an average pore diameter is 29 nm, consistent with a mesoporous material.^22^ In addition, they exhibit a high BET surface area of 498 cm^2^ g^−1^ was determined. After incorporation of Mn_3_O_4_ NPs, the BET surface area and pore volume drop to 201 cm^2^ g^−1^ and 1.28 cm^3^ g^−1^, respectively. This observation can be attributed to Mn_3_O_4_ NPs occupying the space inside the HMCs.

**Fig. 7:**
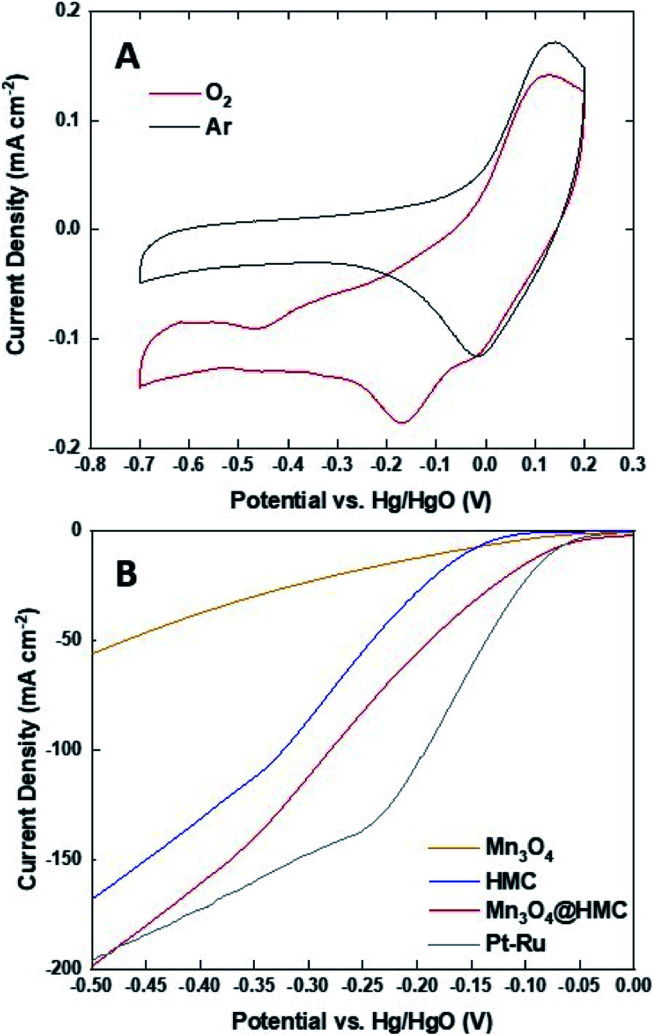
(A) Cyclic voltammograms for Mn_3_O_4_@HMC in Ar- and O_2_-saturated 0.1 M KOH at a scan rate of 5 mV s^−1^; (B) LSV curves obtained in O_2_-saturated 1 M KOH aqueous solution at a scan rate of 5 mV s^−1^.

The ORR activity of Mn_3_O_4_@HMC was first assessed using cyclic voltammetry (CV) in both Ar- and O_2_-saturated 0.1 M KOH aqueous solution using a rotating disk electrode (Fig. 7A). For these measurements, a 0.1 M KOH aqueous electrolyte was used to maximize O_2_ solubility and minimize background current.^32^ The Ar-saturated cyclic voltammogram is featureless and only shows capacitive current; in contrast, the cyclic voltammogram from the O_2_-saturated system shows a cathodic current at −0.17 V. This observation indicates that the ORR reaction occurs on the Mn_3_O_4_@HMC. The ORR performance was subsequently evaluated using standard linear sweep voltammetry (LSV) in 1 M KOH aqueous electrolyte. Electrodes were prepared by impregnating a circular GDL with pristine HMC or Mn_3_O_4_@HMC. LSV curves of pristine HMC, Mn_3_O_4_ NPs, Mn_3_O_4_@HMCs, and Pt–Ru are shown in Fig. 7B. The onset potential (defined as the potential at which the current density reaches 10 mA cm^−2^) for the hybrid material was markedly improved from −0.156 V for the pristine HMC to −0.082 V; this suggests synergistic effects resulting from the combination of HMCs with Mn_3_O_4_ NPs. This performance is comparable to that of Pt–Ru (−0.077 V). In addition, the maximum current density obtained for Mn_3_O_4_@HMC is 198.1 mA cm^−2^ and exceeds that of pristine HMC (*i.e.*, 167.6 mA cm^−2^); it is marginally better than that of the Pt–Ru (*i.e.*, 196.5 mA cm^−2^). Based upon these observations, Mn_3_O_4_@HMC exhibits improved catalytic activity relative to pristine HMC and meets or exceeds the performance of our Pt–Ru benchmark.

**Fig. 8:**
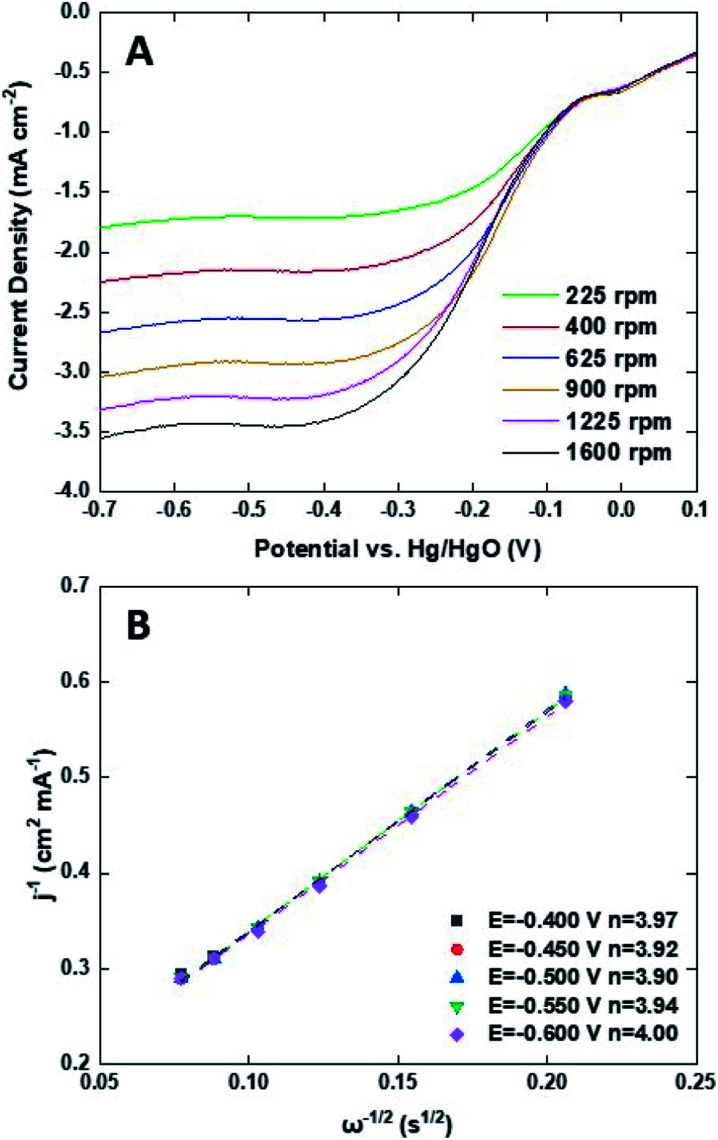
(A) ORR LSV curves for Mn_3_O_4_@HMC in O_2_-saturated 0.1 M KOH at scan rate of 5 mV s^−1^ using RDE; (B) K–L plots for Mn_3_O_4_@HMC extrapolated in the potential range of 0.4–0.6 V.

To investigate the kinetics and catalytic mechanism, LSV was also performed using a Mn_3_O_4_@HMC-coated rotating disk electrode (RDE) at a scan rate of 5 mV s^−1^ and predefined rotation rates (*i.e.*, 225, 400, 625, 900, 1225, and 1600 rpm) in O_2_-saturated 0.1 M KOH electrolyte (Fig. 8A). In Fig. 8A, kinetically controlled (0 to −0.1 V), kinetic-diffusion controlled (−0.1 to −0.3 V), and diffusion controlled (−0.3 to −0.7 V) regions were observed. The Koutecky–Levich (K–L) eqn (1) was used to determine the number of electrons transferred:1
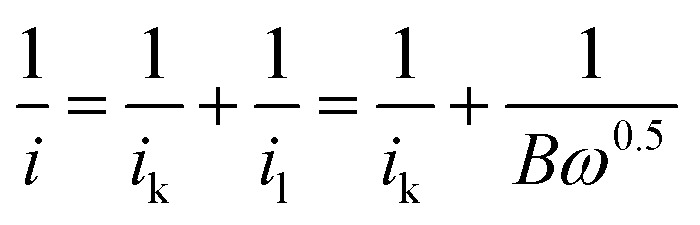

2 *i*_k_ = *nFAkC*_O_2__

3 *B* = 0.62*nAFC*_O_2__*ν*^−1/6^*D*_0_^2/3^

where the measured current (*i*) is related to the kinetic current (*i*_k_) and the diffusion-limiting current (*i*_l_). *ω* is the angular velocity of the RDE, *n* is the number of electrons transferred per O_2_ molecule, *F* is the Faraday constant (96 485 C mol^−1^), *A* is the electrode area (cm^2^), *k* is the electron rate transfer constant, *C*_O_2__ is the saturated O_2_ concentration of the electrolyte (1.2 × 10^−6^ mol cm^−3^), *ν* is the kinematic viscosity of the 0.1 M KOH solution (0.01 cm^2^ s^−1^), and *D*_0_ is the diffusion coefficient of O_2_ in the electrolyte (1.9 × 10^−5^ cm^2^ s^−1^).^33,34^ As the rotation rate was increased, higher (more negative) current densities were observed as a result of faster O_2_ flux to the electrode surface. K–L plots were constructed by plotting *i*^−1^ against *ω*^−0.5^. The number of electrons transferred per O_2_ molecule was obtained from the slope of the fitted lines. The average *n* value in the potential window 0.3–0.7 V is 3.95, suggesting that the Mn_3_O_4_@HMC catalyst predominantly proceeds *via* the four-electron pathway (eqn (6)) rather than the competing alternative two-electron pathway (eqn (4) and (5)).^35^

4 O_2_ + 2H_2_O + 2e^−^ → HO^−^_2_ + OH^−^

5 2HO^−^_2_ → 2OH^−^ + O_2_

6 O_2_ + 2H_2_O + 4e^−^ → 4OH^−^

**Fig. 9:**
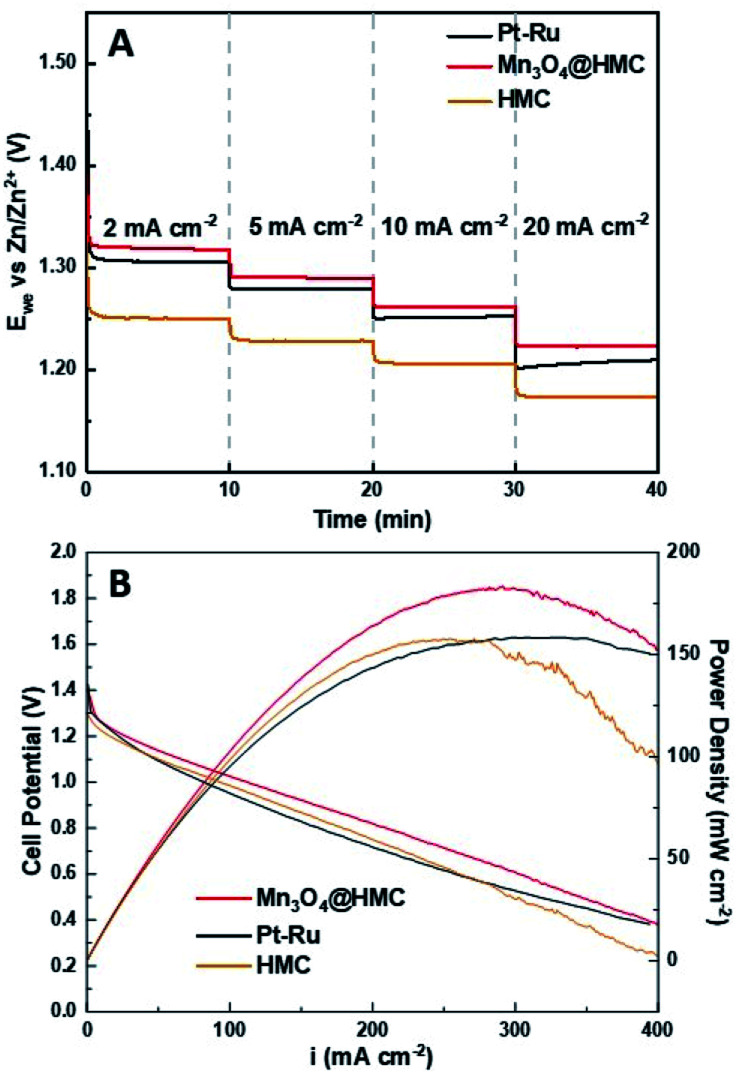
(A) Rate discharge curves; (B) polarization and power density curves for a primary Zn–air battery using Mn_3_O_4_@HMC, pristine HMC, and Pt–Ru as ORR catalysts.

Mn_3_O_4_@HMC and pristine HMC were incorporated into separate primary Zn–air batteries as the air electrode catalyst to evaluate their performance. Discharge rate tests were performed at predefined current densities (2, 5, 10, and 20 mA cm^−2^). In all cases, the rate discharge curves (Fig. 9A) show the discharge potential of the Mn_3_O_4_@HMC was markedly better compared with pristine HMC. Mn_3_O_4_@HMC also outperformed Pt–Ru at the tested current densities, indicating its superior ORR catalytic activity. At 10 and 20 mA cm^−2^, the discharge potentials were 1.26 and 1.22 V, respectively for Mn_3_O_4_@HMC. Our benchmark Pt–Ru catalyst only exhibited 1.25 and 1.20 V discharge potential at 10 and 20 mA cm^−2^, respectively. Furthermore, the performance of the present Mn_3_O_4_@HMC outperforms many transition metal oxide and carbon nanomaterial hybrids (Table S1†). This is also the first time N-doped hollow carbon nanospheres have been combined with transition metal oxide nanoparticles as a Zn–air battery catalyst. Conventional transition metal nanoparticle synthesis often involves high temperature annealing (>300 °C), which is likely to destroy the delicate feature of the HMC (average thickness = 3.8 nm).^36–38^ By implementing the sonication procedure, the potential damage to the HMC active sites was avoided.

Polarization and power density curves are shown in Fig. 9B. They highlight that the maximum power density was improved from 158 mW cm^−2^ to 183 mW cm^−2^ after the incorporation of Mn_3_O_4_ NPs. The maximum power density is much higher than the value obtained for our Pt–Ru benchmark (158 mW cm^−2^) under the same conditions. The hybrid catalyst also exhibits a much lower charge-transfer resistance, evidenced by the smaller size of the semi-circular region of EIS Nyquist plot (Fig. S8†). The excellent catalytic performance can be attributed to the exterior and interior surfaces of the HMCs being decorated with Mn_3_O_4_ NPs. The number of active sites was maximized and any synergistic effects were amplified.

**Fig. 10:**
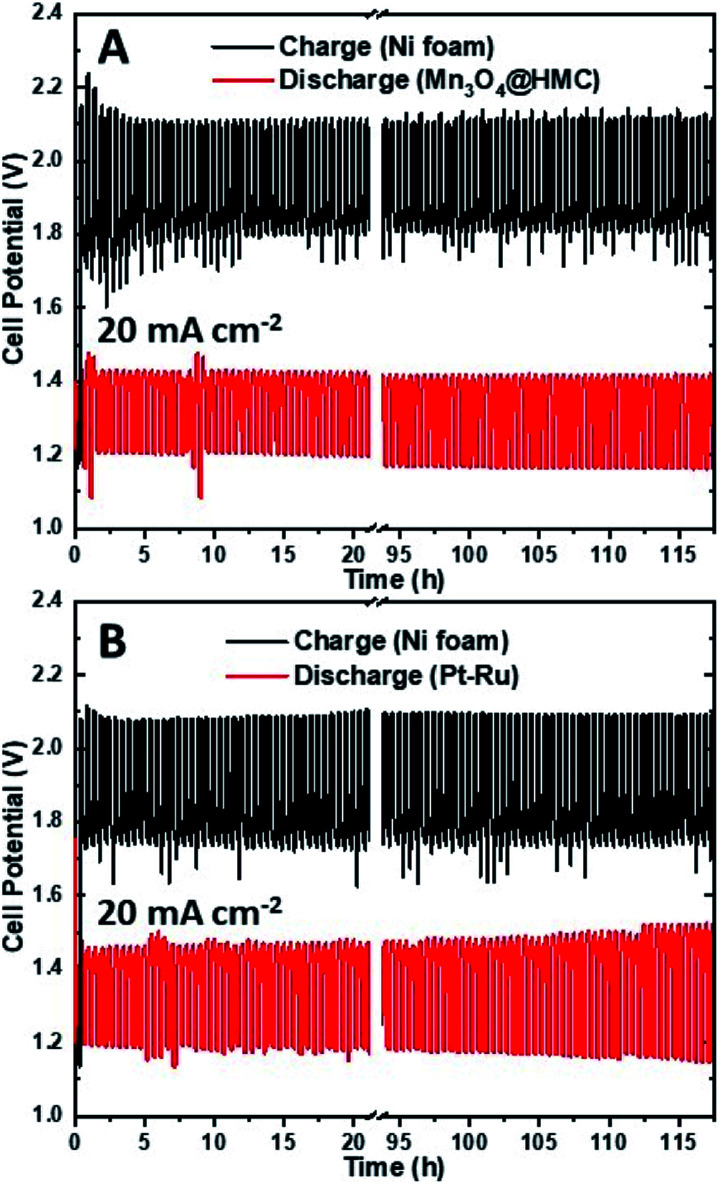
(A) Discharge/charge cycling curves for a three-electrode Zn–air battery using Mn_3_O_4_@HMC on GDL and Ni foam as ORR and OER electrodes, respectively. (B) Discharge/charge cycling curves for a three-electrode Zn–air battery using Pt–Ru on GDL and Ni foam as ORR and OER electrodes, respectively.

The stability of the as-synthesized hybrid material was further investigated by cycling a rechargeable Zn–air battery at 20 mA cm^−2^ using Mn_3_O_4_@HMC on GDL as the ORR electrode. Each cycle was 30 min and 235 cycles (117.5 h) were performed. Ni foam was used as the OER electrode due to its high surface area, good OER catalytic activity, and stability.^39^ A tri-electrode configuration was utilized so that the ORR performance could be evaluated independently of the OER performance. As shown in the discharge/charge curves (Fig. 10A), the HMC hybrid initially exhibits a discharge potential of 1.21 V. After 117.5 h of cycling (235 cycles), the discharge potential decreased slightly to 1.17 V corresponding to a 3.3% loss. A similar tri-electrode battery, using Pt–Ru on GDL as the ORR electrode and Ni foam as OER electrode, was tested under the same conditions (Fig. 10B). The discharge potential for Pt–Ru decreased from 1.19 to 1.14 V, after 117.5 h of cycling, corresponding to a 4.2% change. This shows the comparable long-term durability of the Mn_3_O_4_@HMC compared with Pt–Ru. The morphology of the HMC hybrid was not evaluated after cycling due to the difficulty in separating the hybrid from the GDL.

## Conclusions

Mn_3_O_4_ decorated N-doped hollow mesoporous carbon nanospheres were prepared using a straightforward templated growth/sonication approach. Comparing performance of the hybrid with that of pristine HMCs, we note substantially improved catalytic ORR activity in primary and secondary Zn–air batteries. This synergistic effect, that can reasonably be attributed to Mn–O–C and Mn–N interactions that manifest in the presented XPS analysis, opens the door to the potential of exquisite tuning of catalytic response that could lead to next-generation systems. The primary battery fabricated using Mn_3_O_4_@HMC generated a maximum power density of 183 mW cm^−2^ at a current density of 291 mA cm^−2^. The secondary battery with a three-electrode configuration exhibited a small discharge potential drop from 1.21 to 1.17 V after over 117.5 h of cycling at 20 mA cm^−2^, displaying excellent durability.

## Conflicts of interest

There are no conflicts to declare.

## Supplementary Material

NA-002-D0NA00428F-s001

## References

[cit1] Chu S., Cui Y., Liu N. (2016). Nat. Mater..

[cit2] Roger I., Shipman M. A., Symes M. D. (2017). Nat. Rev. Chem..

[cit3] Sui S., Wang X., Zhou X., Su Y., Riffat S., Jun Liu C. (2017). J. Mater. Chem. A.

[cit4] Cano Z. P., Banham D., Ye S., Hintennach A., Lu J., Fowler M., Chen Z. (2018). Nat. Energy.

[cit5] Zuo W., Li R., Zhou C., Li Y., Xia J., Liu J. (2017). Adv. Sci..

[cit6] Hwang J. Y., Myung S. T., Sun Y. K. (2017). Chem. Soc. Rev..

[cit7] Liu X., Huang J. Q., Zhang Q., Mai L. (2017). Adv. Mater..

[cit8] Fu J., Cano Z. P., Park M. G., Yu A., Fowler M., Chen Z. (2017). Adv. Mater..

[cit9] Girishkumar G., McCloskey B., Luntz A. C., Swanson S., Wilcke W. (2010). J. Phys. Chem. Lett..

[cit10] Wang Y., Liu B., Li Q., Cartmell S., Ferrara S., Deng Z. D., Xiao J. (2015). J. Power Sources.

[cit11] Pan J., Xu Y. Y., Yang H., Dong Z., Liu H., Xia B. Y. (2018). Adv. Sci..

[cit12] Li Y., Lu J. (2017). ACS Energy Lett..

[cit13] Zhang W., Lai W., Cao R. (2017). Chem. Rev..

[cit14] Cheng F., Chen J. (2012). Chem. Soc. Rev..

[cit15] Liu M., Song Y., He S., Tjiu W. W., Pan J., Xia Y. Y., Liu T. (2014). ACS Appl. Mater. Interfaces.

[cit16] Wang H., Keum J. K., Hiltner A., Baer E., Freeman B., Rozanski A., Galeski A. (2009). Science.

[cit17] Qu L., Liu Y., Baek J.-B., Dai L. (2010). ACS Nano.

[cit18] Ren M., Zhang J., Tour J. M. (2019). ACS Appl. Energy Mater..

[cit19] Cheng F., Su Y., Liang J., Tao Z., Chen J. (2010). Chem. Mater..

[cit20] Koza J. A., He Z., Miller A. S., Switzer J. A. (2012). Chem. Mater..

[cit21] Li L., Yang J., Yang H., Zhang L., Shao J., Huang W., Liu B., Dong X. (2018). ACS Appl. Energy Mater..

[cit22] Hadidi L., Davari E., Iqbal M., Purkait T. K., Ivey D. G., Veinot J. G. C. (2015). Nanoscale.

[cit23] Dasog M., Smith L. F., Purkait T. K., Veinot J. G. C. (2013). Chem. Commun..

[cit24] Shirley D. A. (1972). Phys. Rev. B: Solid State.

[cit25] Xiong M., Clark M. P., Labbe M., Ivey D. G. (2018). J. Power Sources.

[cit26] Wu T.-H., Hesp D., Dhanak V., Collins C., Braga F., Hardwick L. J., Hu C.-C. (2015). J. Mater. Chem. A.

[cit27] Lee J. W., Hall A. S., Kim J. D., Mallouk T. E. (2012). Chem. Mater..

[cit28] Gorlin Y., Jaramillo T. F. (2010). J. Am. Chem. Soc..

[cit29] Chigane M., Ishikawa M., Izaki M. (2001). J. Electrochem. Soc..

[cit30] Yu X., Fan H., Liu Y., Shi Z., Jin Z. (2014). Langmuir.

[cit31] Dubal D. P., Dhawale D. S., Salunkhe R. R., Pawar S. M., Lokhande C. D. (2010). Appl. Surf. Sci..

[cit32] Xing W. , Yin G. and Zhang J., Rotating Electrode Methods and Oxygen Reduction Electrocatalysts, Elsevier B.V., 2014

[cit33] Zhang J., Zhao Z., Xia Z., Dai L. (2015). Nat. Nanotechnol..

[cit34] Paulus U. A., Schmidt T. J., Gasteiger H. A., Behm R. J. (2001). J. Electroanal. Chem..

[cit35] Wu Z. S., Yang S., Sun Y., Parvez K., Feng X., Müllen K. (2012). J. Am. Chem. Soc..

[cit36] Laurent S., Forge D., Port M., Roch A., Robic C., Vander Elst L., Muller R. N. (2008). Chem. Rev..

[cit37] Vijayakumar S., Kiruthika Ponnalagi A., Nagamuthu S., Muralidharan G. (2013). Electrochim. Acta.

[cit38] Chen L., Sun L. J., Luan F., Liang Y., Li Y., Liu X. X. (2010). J. Power Sources.

[cit39] Zhang W., Li D., Zhang L., She X., Yang D. (2019). J. Energy Chem..

