# Supplementary material for: Mn3O4 nanoparticle-decorated hollow mesoporous carbon spheres as an efficient catalyst for oxygen reduction reaction in Zn–air batteries
Source: Nanoscale Adv. 2020 Jun 19;2(8):3367–74. doi: 10.1039/d0na00428f (PMC9418335; doi:10.1039/d0na00428f)
Supplement: NA-002-D0NA00428F-s001 [file NA-002-D0NA00428F-s001.pdf]

Electronic Supplementary Material (ESI) for Nanoscale Advances.
This journal is © The Royal Society of Chemistry 2020

Electronic Supplementary Information

Mn_3O_4 Nanoparticle-Decorated Hollow Mesoporous Carbon Spheres as an Efficient Catalyst for Oxygen Reduction Reaction in Zn-Air Batteries.

Yingjie He[a], Drew Aasen[b], Haoyang Yu[a], Matthew Labbe[b], Douglas G. Ivey*[b], and Jonathan G.C. Veinot *[a]

[a] Department of Chemistry, University of Alberta, 11227 Saskatchewan Drive, Edmonton, Alberta, Canada T6G 2G2

[b] Department of Chemical and Materials Engineering, University of Alberta, 9211 116 St, Edmonton, Alberta, Canada T6G 1H9

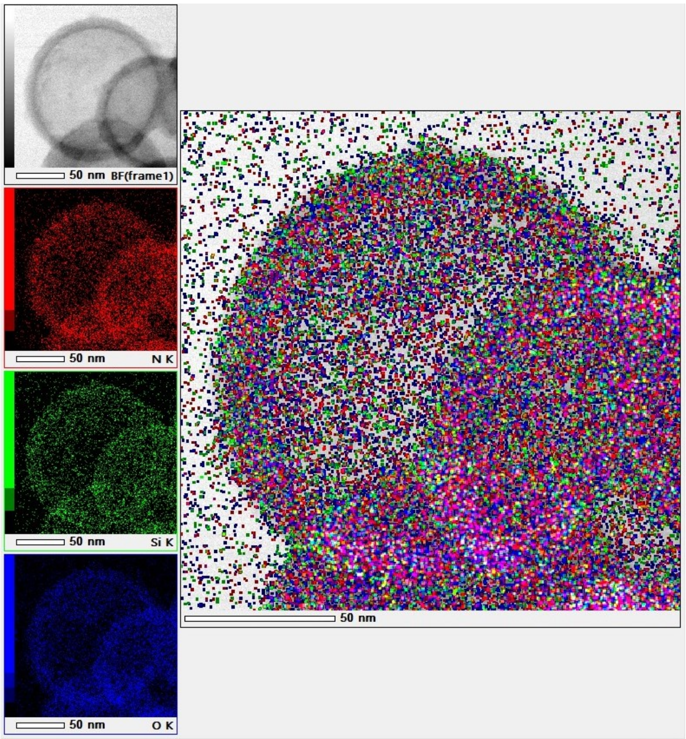

Fig. S1. Elemental mapping of HMCs.

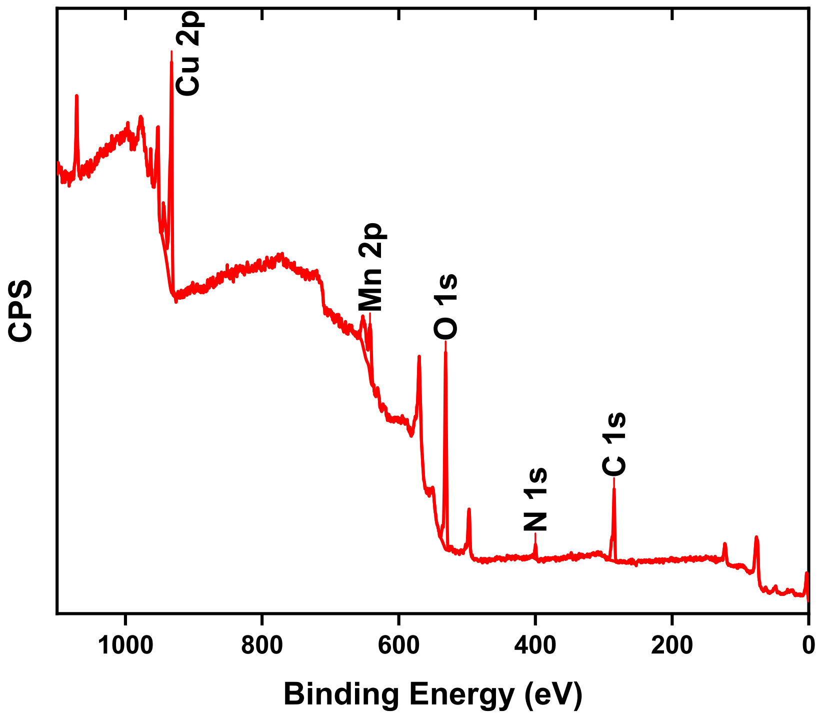

Fig. S2 XPS survey spectrum for Mn_3O_4@HMC.

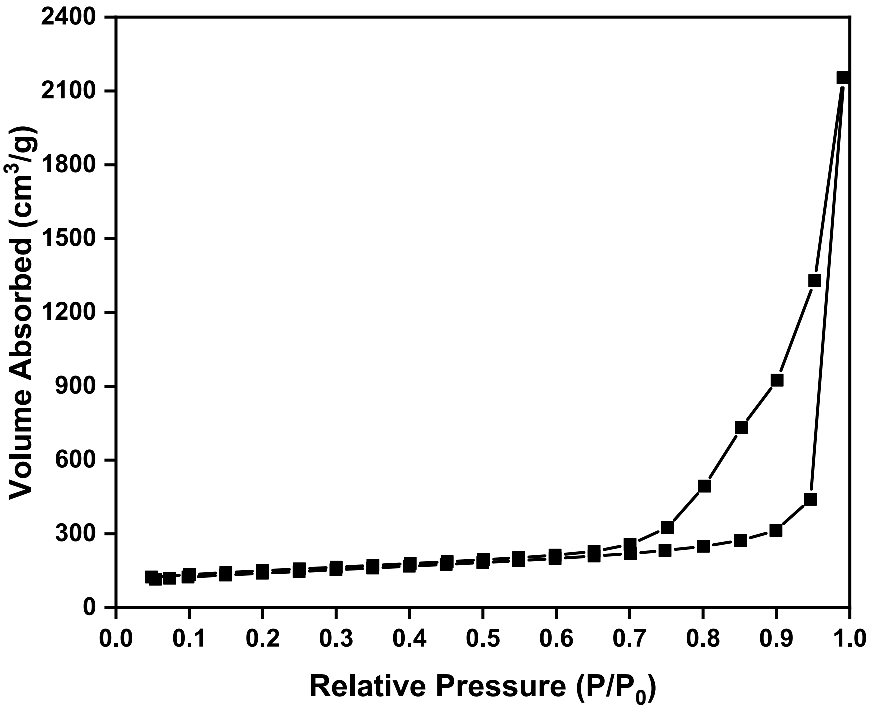

Fig. S3 Nitrogen adsorption-desorption isotherms for purified hollow mesoporous carbon spheres (HMC).

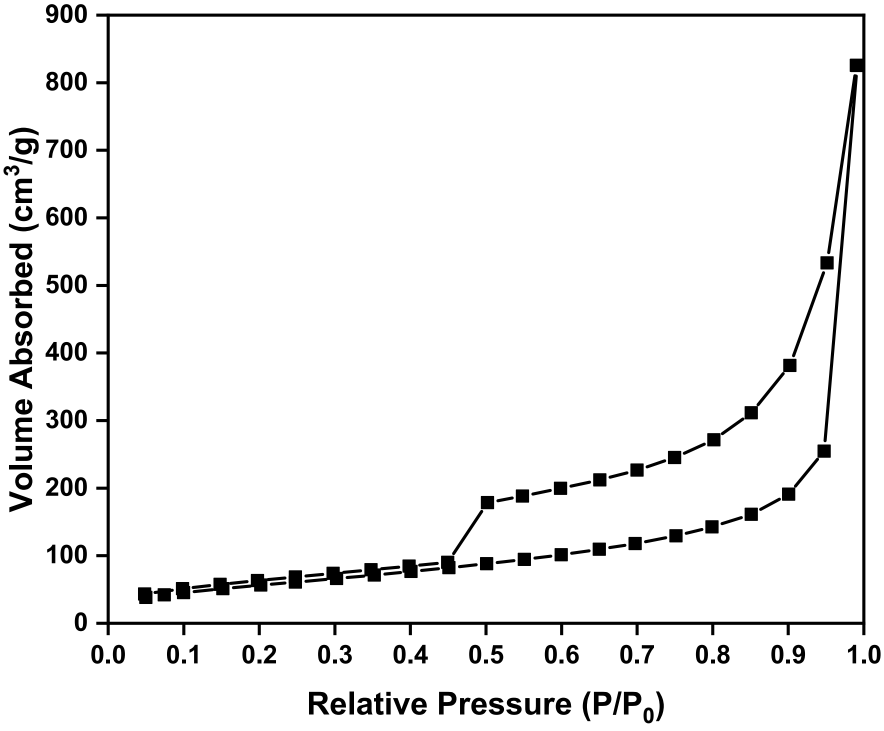

Fig. S4 Nitrogen adsorption-desorption isotherms for the Mn_3O_4 NP decorated hollow mesoporous carbon spheres (HMC@Mn_3O_4).

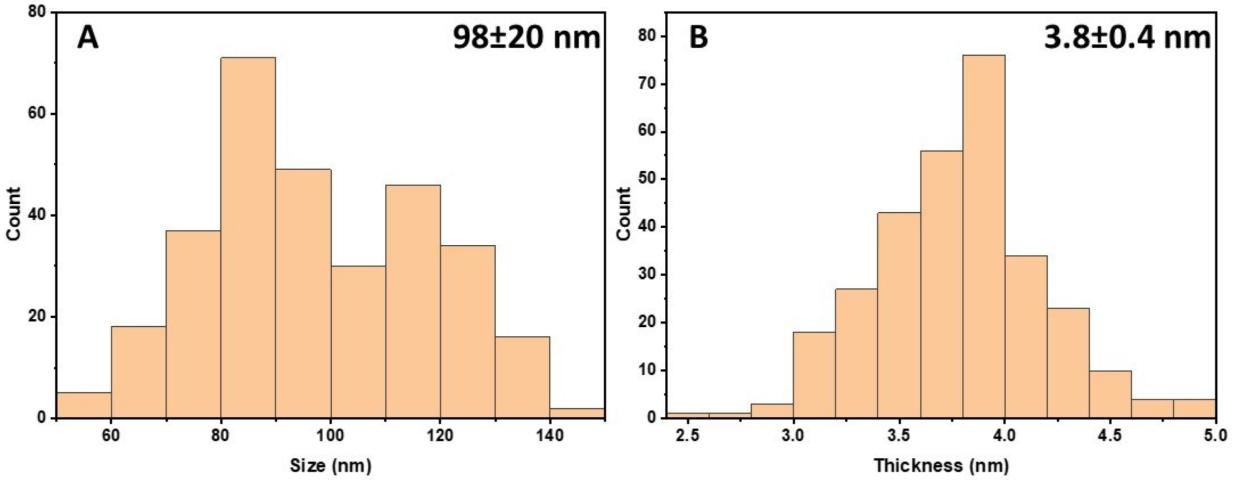

Fig. S5 (A) Size distribution of HMCs; (B) thickness distribution of HMCs.

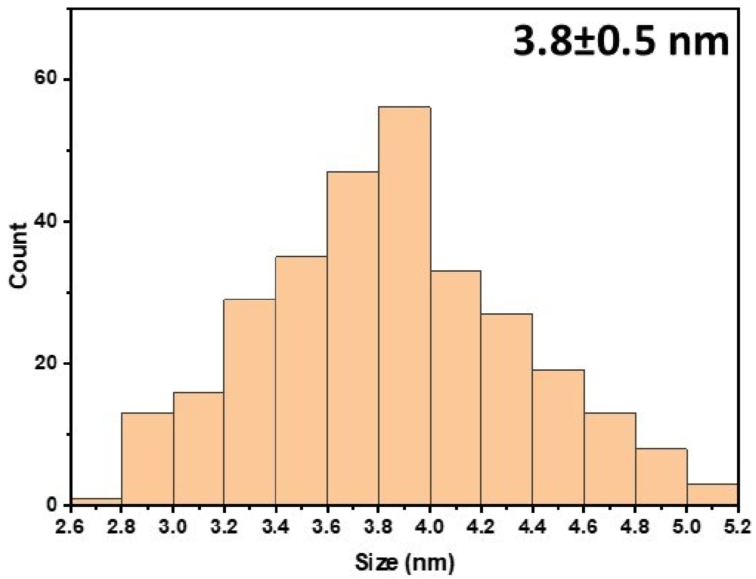

Fig. S6 Size distribution of Mn_3O_4 nanoparticles.

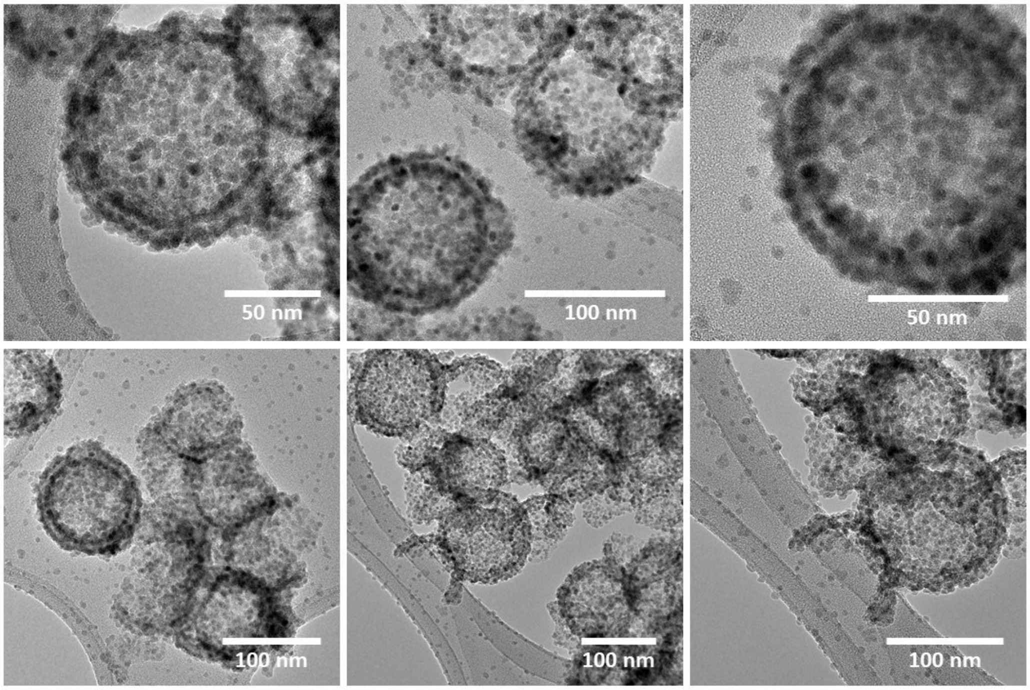

Fig. S7 Additional TEM images of Mn_3O_4@HMC.

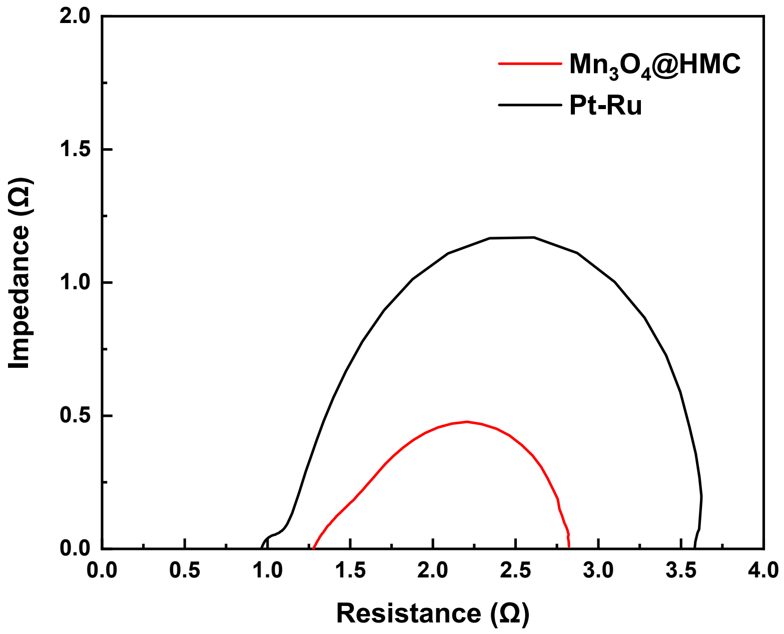

Fig. S8 Electrochemical impedance spectra of Mn_3O_4@HMC and Pt-Ru.

Table S1. Performance Comparison of Mn_3O_4@HMC with Other Zn-Air Battery Catalyst in Literature

Notable Catalysts from Literature	Catalyst Type	Electrolyte	Discharge Potential (V) @ i (mA cm^{-2})	Cyclability	Ref‡
Mn_3O_4@HMC	Transition Metal Oxide and Carbon Hybrid	6 M KOH + 0.25 M ZnO	1.22@20	40 mV drop, 235 cycles, 20 mA cm^{-2}	This work
Mn_3O_4/N-CNT	Transition Metal Oxide and Carbon Hybrid	6 M KOH + 0.25 M ZnO	1.21@20	100 mV drop, 100 cycles, 10 mA cm^{-2}	1
Mn_3O_4 QD/N-p-MCNT	Transition Metal Oxide and Carbon Hybrid	6 M KOH + 0.2 M $Zn(CH_3COO)_2$	~1.1@20	Stable. 100 h, 20 mA cm^{-2}	2
Co_3O_4/N-CNT on Stainless Steel Mesh	Transition Metal Oxide and Carbon Hybrid	Cellulose Gelled w/ 6 M KOH	1.2@25	Stable, 60 cycles, 25 mA cm^{-2}	3
$Fe_{0.5}Co_{0.5}O_x$/N-rGO	Transition Metal Oxide and Carbon Hybrid	6 M KOH + 0.2 M $ZnCl_2$	1.21@10	50 mV drop, 60 cycles, 10 mA cm^{-2}	4
Fe-N-CNN	Metal Organic Framework	6 M KOH	1.21@20	N/A – Not cycled	5
$MnCo_2O_4$/CNT	Transition Metal Oxide and Carbon Hybrid	30 wt% KOH + 20 g L^{-1} $ZnCl_2$	1.20@10	200 mV drop, 70 cycles, 15 mA cm^{-2}	6
MnO_2 on Carbon Paper	Transition Metal Oxide on Carbon Support	6 M KOH + 20 g L^{-1} $ZnCl_2$	1.20@15	20 mV drop, 350 cycles, 15 mA cm^{-2}	7
Co_4N/CNW/CC	Transition Metal Oxide and Carbon Hybrid	6 M KOH + 0.2 M Zn Acetate	1.15@10	Stable over 400 cycles	8
Fe@N-C	N-C Encapsulated Transition Metal	6 M KOH + 0.2 M Zn Acetate	1.25@10	125 mV drop, 200 cycles, 10 mA cm^{-2}	9

References

1 D. Aasen, M. Clark and D. G. Ivey, *Batter. Supercaps*, 2019, **2**, 882–893.

2 Z. Huang, X. Qin, X. Gu, G. Li, Y. Mu, N. Wang, K. Ithisuphalap, H. Wang, Z. Guo, Z. Shi, G. Wu and M. Shao, *ACS Appl. Mater. Interfaces*, 2018, **10**, 23900–23909.

3 J. Fu, F. M. Hassan, J. Li, D. U. Lee, A. R. Ghannoum, G. Lui, M. A. Hoque and Z. Chen, *Adv. Mater.*, 2016, **28**, 6421–6428.

4 L. Wei, H. E. Karahan, S. Zhai, H. Liu, X. Chen, Z. Zhou, Y. Lei, Z. Liu and Y. Chen, *Adv. Mater.*, 2017, **29**, 1701410.

5 F. Li, H. Li, X. Liu, L. Wang, Y. Lu and X. Hu, *Chem. – A Eur. J.*, 2018, **25**, chem.201804643.

6 X. Ge, Y. Liu, F. W. T. Goh, T. S. A. Hor, Y. Zong, P. Xiao, Z. Zhang, S. H. Lim, B. Li, X. Wang and Z. Liu, *ACS Appl. Mater. Interfaces*, 2014, **6**, 12684–12691.

7 A. Sumboja, X. Ge, F. W. T. Goh, B. Li, D. Geng, T. S. A. Hor, Y. Zong and Z. Liu, *Chempluschem*,

2015, **80**, 1341–1346.

8 F. Meng, H. Zhong, D. Bao, J. Yan and X. Zhang, *J. Am. Chem. Soc.*, 2016, **138**, 10226–10231.

9 J. Wang, H. Wu, D. Gao, S. Miao, G. Wang and X. Bao, *Nano Energy*, 2015, **13**, 387–396.
